# Commentary: Reconstruction of Pharyngolaryngeal Defects with the Ileocolon Free Flap: A Comprehensive Review and How to Optimize Outcomes

**DOI:** 10.1055/s-0043-1777220

**Published:** 2024-03-04

**Authors:** Joseph M. Escandón

**Affiliations:** 1 Division of Plastic and Reconstructive Surgery, Strong Memorial Hospital, University of Rochester Medical Center, Rochester, New York

Reconstruction of Pharyngolaryngeal Defects with the Ileocolon Free Flap: A Comprehensive Review and How to Optimize Outcomes

Several reconstructive methods have been reported to restore the continuity of the aerodigestive tract following resection of pharyngeal and hypopharyngeal cancers. However, high complication rates have been reported after voice prosthesis insertion. In this setting, the ileocolon free flap (ICFF) offers a tubularized flap for reconstruction of the hypopharynx while providing a natural phonation tube. Herein, we systematically reviewed the current evidence on the use of the ICFF for reconstruction of the aerodigestive tract. A systematic literature search was conducted across PubMed MEDLINE, Web of Science, ScienceDirect, Scopus, and Ovid MEDLINE(R). Data on the technical considerations and surgical and functional outcomes were extracted. Twenty-one studies were included. The mean age and follow-up were 54.65 years and 24.72 months, respectively. An isoperistaltic or antiperistaltic standard ICFF, patch flap, or chimeric seromuscular-ICFF can be used depending on the patients' needs. The seromuscular chimeric flap is useful to augment the closure of the distal anastomotic site. The maximum phonation time, frequency, and sound pressure level (dB) were higher with ileal segments of 7 to 15 cm. The incidence of postoperative leakage ranged from 0 to 13.3%, and the majority occurred at the coloesophageal junction. The revision rate of the microanastomosis ranged from 0 to 16.6%. The ICFF provides a reliable and versatile alternative for reconstruction of middle-size defects of the aerodigestive tract. Its three-dimensional configuration and functional anatomy encourage early speech and deglutition without a prosthetic valve and minimal donor-site morbidity.



